# Incentive strategy of safe and intelligent production in assembled steel plants from the perspective of evolutionary game

**DOI:** 10.1038/s41598-023-29097-8

**Published:** 2023-02-04

**Authors:** Yinglin Wang, Leqi Chen, Yulong Li, Caiquan Chen, Jiaxin Zhuang

**Affiliations:** 1 School of Public Affairs, Xiamen University, Xiamen, 361005 China; 2 School of Transportation and Civil Engineering, Fujian Agriculture and Forestry University, Fuzhou, 350100 China; 3 CSCEC Strait Construction and Development Co., Ltd, Fuzhou, 350015 China

**Keywords:** Civil engineering, Energy infrastructure

## Abstract

Due to the numerous cross-operations and poor information communication, it is easy to cause production safety accidents in traditional assembled steel plants. The transformation and upgrading of smart production in the assembly steel plants is helpful to improve the efficiency of safety management. In order to effectively reduce the safety risks in the production of assembled steel components, this paper integrates policy incentives and safety supervision, constructs an evolutionary game model between the government and assembled steel producers, and analyzes the strategic evolution rules and stability conditions of stakeholders through the replication dynamics equation. Moreover, based on the empirical simulation of the Fuzhou X Steel Structure Plant project, the effectiveness of the evolutionary model incentive strategy setting is verified. The results show that whether an assembled steel plants adopt a smart management strategy or not is influenced by the government's incentive subsidy mechanism, penalty mechanism, the benefits and costs generated by traditional/ smart management, the probability and loss of safety accidents and other factors. The conclusion is important for upgrading the safety management mode, improving the safety production efficiency and constructing the safety supervision system of the assembled steel smart plant.

## Introduction

Assembled building with steel-structure is a high quality and green building mode. Compared with the traditional pouring concrete building, it has the advantages of short construction period, recyclable structure, environmental protection, high quality and safety level. China achieved 6.2% of new constructions with assembled steel-structure buildings in 2020, with a 33% increase in floor space compared with 2019^1^. However, there are many problems in traditional assembly steel structure production, such as poor information communication, untimely management coordination, lack of skilled workers, and many equipment cross-operation. The above problems are prone to form safety risks and thus lead to accidents. The intelligent operation platform and security management system are built on the basis of big data. It integrates plant data information through integrated software, IoT (Internet of things) devices and smartphone terminal, which helps to supervise project hidden danger, personnel and equipment and other dangerous sources in the whole process and realize the standardization and intelligence of safety production in assembly steel plants.

In order to effectively reduce the safety risks in the production of assembled steel components, this paper combs the smart production management process of assembled steel structures and demonstrates the management mode, information characteristics and safety production effectiveness of the smart plant. Since the introduction of smart construction technology will increase the initial investment cost of the project, many steel manufacturers are not very motivated to upgrade their industry. In this case, policy guidance is significant to stimulate assembled steel manufacturers to participate in the transformation and upgrading of smart production. Based on this, this paper discusses the evolutionary path of the safety production strategies of assembled steel manufacturers under the dual mechanisms of smart production subsidies and safety supervision and punishment strategies by the government.

## Literature review

### Industry 3.0 to Industry 4.0 changes in steel plants

After the three major industrial revolutions, the globalization division of labor accelerated the flow and allocation of production factors. The changing market direction and personalized product demand have put forward higher requirements for enterprises' response time and flexible operation capability. The world has entered an era of intensive innovation and industrial transformation^2^. In this context, the 4.0 industrial revolution led by intelligent manufacturing is coming quietly. In fact, Industry 3.0 has already achieved the integration of manufacturing and digital technology through electronic information technology. For example, in the era of Industry 3.0, computer numerical control (CNC) machine tool technology was applied in the product production process^3^ and simulation modeling and physical test virtualization technologies were used in the design process of digital plant components^4^. Compared with Industry 3.0, Industry 4.0 integrates physical and virtual digital technologies to realize the digitalization of the whole process of product production^5^. Industry 4.0 uses the information interaction characteristics of the Internet to propose the concept of smart plants, which further breaks through the information barrier between various organizations and equipment^6^. It not only realizes intelligent manufacturing in the manufacturing stage, but also realizes all-round intelligence in the production decision-making and management stage, such as raw material procurement, production scheduling, delivery arrangement and other links.

As an important economic pillar of the manufacturing industry, the intellectualization of the steel structure industry lags far behind other manufacturing industries. In the traditional steel structure production process, many steps require manual operation. Taking the welding step as an example, Hajikaimi et al.^7^ pointed out that the safety in the welding process depends not only on the appropriate equipment but also to a large extent on the proficiency of the welders. However, in the production process, the enterprise cannot guarantee that every worker can strictly implement the safety production process at all times, which leads to the generation of safety risks. In order to adapt to the changing rhythm of industry 4.0 era, the traditional steel structure manufacturing industry must update and adjust the existing working model to achieve intelligent transformation^8^. Cho et al.^9^ pointed out that in the process of assembly, high-rise operation is very dangerous and requires highly professional workers, while the application scope of building automation in the project implementation process is very limited. Therefore, they defined each part of steel as different elements based on analytic hierarchy process and carried out quantitative analysis, and designed an automatic system of steel frame manufacturing. Zhao et al.^10^ established a virtual library to meet customer requirements and formed an intelligent process management system to solve the problems of complicated manufacturing process and long construction period caused by randomness and uncertainty in the traditional steel production process. Wang et al.^11^ pointed out that steel structure enterprises generally have problems such as low information level, high production cost and serious resource waste, so they proposed a manufacturing execution system (MES) based on cyber physical systems to improve the informationization level of manufacturing plants. Tavares et al.^12^ proposed a welding unit that can be connected in series with production links. It uses BIM technology to automatically arrange and delegate tasks to welding manipulators and operators, thus optimizing product quality and reducing production costs on the production line. In summary, the current research related to smart construction in the construction industry focuses on BIM-based steel structure research^13^, BIM-based assembly building research^14,15^, and smart technology and construction-related research^16,17^. Research on smart production of prefabricated steel structure mainly focuses on the application of BIM technology, IoT and other intelligent technologies in steel structure, assembled building design^18^, assembled construction^19^ and management^20^.

Intelligent management of assembled steel structure production process is of great significance to improve safety efficiency. Under the background of industry 4.0, intelligent transformation and upgrading of the construction industry is the only way to adapt to the development of informationization^21^. Compared with other manufacturing industries, the transformation and upgrading of intelligent technology in the construction industry is more difficult. Faced with the high cost of intelligent equipment investment and the uncertainty of income, many assembled steel pants balk at the decision of smart production transformation.

### Application of evolutionary game

The research framework of classical game theory is based on the assumption that the game subject has perfect judgment. This assumption can only explain the reasons for the cooperation between construction enterprises, but cannot reveal the long-term cooperative behavior rules and the stable and sustainable development path of enterprises. Based on the information asymmetry and the heterogeneous social preferences of game participants, the cooperation strategies displayed by game decision makers are not completely rational in practice^22^. Different from the assumption of complete rationality of actors in classical game theory, evolutionary game theory proposes that decision makers involved in the game are bounded rationality. Specifically, in the process of pursuing maximum benefits, participants in evolutionary game can learn through observation, imitation, trial and error, and can continuously adjust their decisions to improve the effectiveness of the game. Based on this, more and more researchers tend to use evolutionary game theory to analyze and explain game phenomena in real life^23,24^.

In engineering construction, the introduction of evolutionary game theory is helpful to reflect the diversity and complexity of behavioral strategies of economic activity subjects more truly and objectively^25^. At present, evolutionary game has been widely used by scholars in the conflict, coordination and interaction among stakeholders in many engineering construction fields, such as PPP project construction and operation^26^, the establishment of waste incineration power plant^27^ and the green innovation of rail transit project^28^. With the promotion of Industry 4.0, how to accelerate the landing of steel structure intelligent factory and promote intelligent transformation is a question worth exploring. At this stage, the research on assembled construction focuses more on the advantages and disadvantages of assembled construction^29^, while few scholars have studied the intelligent safety production of steel plants. Because smart plants have different stakeholders, it is appropriate to choose evolutionary game as the research method.

In summary, the research contributions of this paper are mainly reflected in the following aspects: first, to solve the concerns of assembled steel plants in the intelligent transformation process, this paper demonstrates the safety and feasibility of intelligent manufacturing by sorting out the process flow of smart production management. Then, based on the evolutionary game theory, the benefit function of government and assembled steel plant based on the safety production efficiency is constructed, and the evolution path of factory implementing smart safety production decision-making under different incentive policies is analyzed. Finally, based on the empirical simulation of Fuzhou X Steel Structure Plant project, the paper puts forward decision-making suggestions for the government to formulate reasonable subsidy strategies to accelerate the intelligent transformation of traditional steel structure plants and explore the development mechanism to promote intelligent manufacturing.

### Smart production management process for assembled steel structures

The steel structure production process has the following characteristics: first, the manufacturer needs to determine the method of processing, assembling and welding according to the different combinations and stress conditions of the complex components, and carry out the process test to ensure the quality of the finished products. Secondly, the gauges used in the fabrication, installation, acceptance and project construction of steel structures should be identified according to the unified measurement standards to ensure that the finished products have the agreed accuracy level. In particular, the steel structure projects of high-rise civil buildings has the characteristics of long construction period and large environmental temperature difference. In order to avoid the deviation of gauges, it is necessary for the production unit to calculate the correction value strictly according to the actual situation of temperature to ensure the accuracy of finished product size^30^. Therefore, the safety management of the assembly steel plant needs to meet the requirements of safe production management and safe storage management.

The safety management mode of traditional plants is relatively isolated from the comprehensive management of project production. At the present stage, the following deficiencies exist in the production safety management of traditional steel plants: firstly, there are many internal production activities and large manpower requirements. Complicated production procedures and massive document sorting work lead to the plant production information update lag. Under such conditions, it is still difficult to reduce the probability of safety accidents even if the supervisors carry out regular on-site testing^31^. Secondly, the layout of production work has strong limitations. In the traditional steel structure production process, the material storage point is far away from the material processing point, which is not conducive to construction. In addition, improper storage of materials also breeds safety hazards. The current management model is mainly based on manpower and lacks real-time monitoring of the equipment site, which results in a large amount of wasted resources and reduces the production efficiency of the steel plant.

Safety management is one of the important elements of smart plant management. Under the background of the rapid development of modern network technology, the design of safety management system based on Wi-Fi network can promote the construction of automatic positioning and intelligent monitoring, thus providing guarantee for the improvement of plant safety management efficiency^32^. China Construction Steel Structure Co., Ltd has established the first digital plant based on industrial internet platform in China's construction steel structure field. Based on intelligent manufacturing, equipment utilization and production efficiency have been greatly improved, and operating costs, labor utilization and unit energy consumption have been reduced. With the reduction of personnel and the strengthening of integration, the probability of steel component production safety accidents is also reduced. It is necessary for managers to establish an enterprise production information system with the support of modern information technologies such as cloud computing, IoT, network communication and mobile application^33^, so as to solidify the operation experience of employees into systematic expert knowledge and provide real-time help for front-line production personnel.

**Figure 1:**
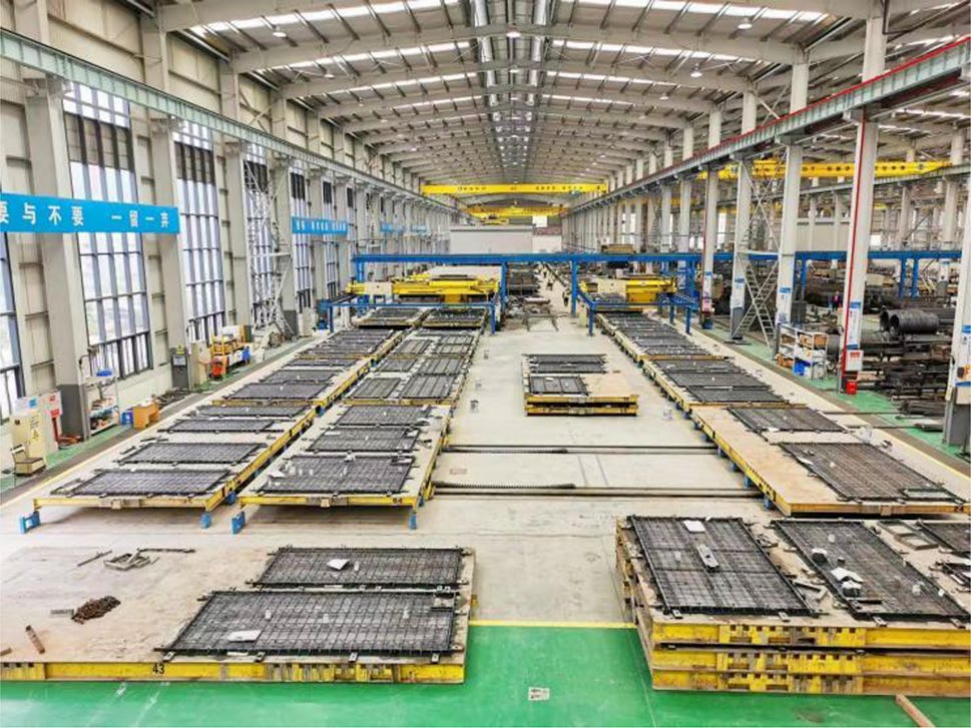
Intelligent manufacturing site.

**Figure 2:**
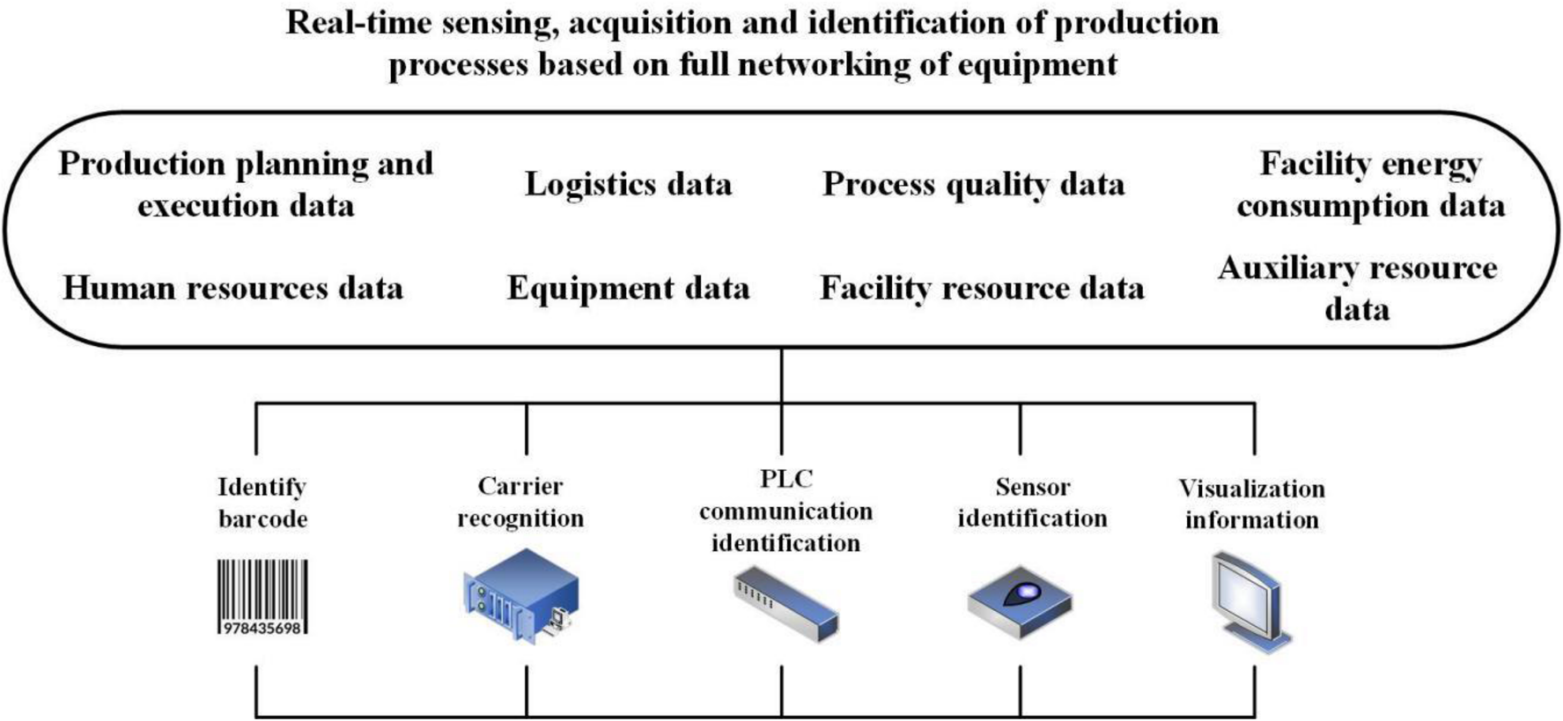
Production process based on equipment full network.

The smart plant information system helps guide the safe and smooth operation of plant facilities, promote safe production and improve manufacturing efficiency. Figure 1 shows the smart factory site. Intelligent manufacturing mainly applies high-grade CNC (Computerized Numerical Control) machine tools and industrial robots, intelligent sensing and control equipment, intelligent testing and assembly equipment, intelligent logistics and storage equipment, intelligent processing units. The construction of smart plant's information system mainly relies on big data analysis technology and guarantees the reliability of data based on the safety management system^34^. Figure 2 shows the real-time perception, acquisition and identification process based on equipment full network production. The establishment of automatic production process data collection and analysis system can realize automatic uploading of site data such as production progress, site operation, quality inspection, equipment status and material transmission. The system can generate intuitive data through large data screens and monthly reports, so as to ensure the timeliness of information and realize the visual management of projects^35^.

The principle of intelligent construction information sharing is based on technologies such as the IoT, integrating physical systems into computer systems through software, and establishing links between enterprises to carry out real-time data sharing^36^. Cloud computing technology helps store massive real-time data of industrial manufacturing from diverse sources, analyze individual customer needs, guide and optimize production and inventory management^37^. In manufacturing systems, machine learning technologies can extract valuable information from big data to satisfy managers monitoring defects, detecting failures, and predicting future needs^38^. In addition, the security risk pre-control system helps to prevent data loss^39^, warn of emergencies, and promptly report accident hazards in the plant. The intelligent production system is based on the data generated by the information exchange among stakeholders^40^. With the help of the IoT and information simulation technology, the intelligent production system can control the risk source in advance and formulate preventive measures to efficiently deal with the report of security accidents and attempted incidents^41^.

## Methodology

### Construction of management strategy model and parameter assumption of assembled steel smart plant

Intelligent management of assembled steel structure production process is of great importance to improve safety efficiency. However, the investment cost of intelligent management makes many enterprises flinch. In order to fully release the dividends of the big data era, it is necessary to take appropriate incentives to promote the intelligent upgrading of safe production of assembled steel structures. Therefore, this paper incorporates government departments and assembled steel plants into the game relationship of the incentive model, assuming that both are finite rational evolutionary game subjects that will continuously optimize their strategy choices in the repeated game process. The strategy choice of the government department includes incentive or non-incentive, and there exist two strategies: smart production or traditional production.

Assumption 1: The government department provides incentives to the assembled steel plants with probability *q* (0 ≤ *q* ≤ 1). *G* denotes the subsidies based on the construction of the incentive intelligent information platform, and *w* denotes the corresponding penalties set for the generated safety accidents under the incentive policy. The assembled steel plant has *p* (0 ≤ *p* ≤ 1) probability to choose the smart plant to enhance the safety production management. θ_1_ denotes the probability of safety accidents in the production of smart plants. θ_2_ denotes the probability of safety accidents in the production of traditional steel plants. The loss to be paid by the assembled steel plant in case of safety accident is *D*.

Assumption 2: The benefits and costs generated by the assembled steel smart plant project are *R*_1_ and *C*_1_ respectively, and those generated by the traditional steel plant project are *R*_2_ and *C*_2_, respectively. When government departments choose to incentivize assembled steel plants, the increase in government credibility brought by safe production is *S*_1_, and the loss of credibility generated by a safety accident is *S*_2_.

The meanings of parameters involved in the model are shown in Table 1.

**Table 1:** Description of model parameters.

Symbols	Parameter description
$S_{1}$	Increased credibility when governments choose to incentivize safe production in assembled steel plants
$S_{2}$	Loss of government credibility arising from safety accidents
$G$	Subsidy based on the construction of policy incentive intelligent information platform
$w$	Penalties for safety accidents under the incentive policy are set accordingly
$R_{1}$	Benefits of assembled steel smart plant project
$R_{2}$	Benefits of traditional steel plant project
$C_{1}$	Costs incurred by the assembled steel smart plant project
$C_{2}$	Costs incurred by traditional steel plant project
$\theta_{1}$	The probability of safety accidents in the production of assembled steel smart plant
$\theta_{2}$	The probability of safety accidents in the production of traditional steel plants
$D$	Loss to be paid by the assembled steel plant in case of a safety accident

Based on the above assumptions and analysis, an evolutionary game model between the government and the assembled steel plant is constructed, and the obtained benefit matrix is shown in Table 2.

**Table 2:** Game payment matrix between government (A_1_) and assembly steel plant (A_2_).

*A*_*2*_ /*A*_*1*_	$b_{H} \left( p \right)$	$b_{L} \left( {1 - p} \right)$
$b_{Y} \left( q \right)$	$(1 - \theta_{1} )S_{1} - G - \theta_{1} S_{2} + \theta_{1} w,R_{1} - C_{1} + G - \theta_{1} D - \theta_{1} w$	$(1 - \theta_{2} )S_{1} - \theta_{2} S_{2} + \theta_{2} w,R_{2} - C_{2} - \theta_{2} D - \theta_{2} w$
$b_{N} \left( {1 - q} \right)$	$- \theta_{1} S_{2} ,R_{1} - C_{1} - \theta_{1} D$	$- \theta_{2} S_{2} ,R_{2} - C_{2} - \theta_{2} D$

When the government chooses to incentivize, the expected return is *u*_11_, while the expected return for choosing not to incentivize is *u*_12_, so the average return is *ū*_1_. The corresponding expression is as follows:

1 $$u_{11} = p\left[ {(1 - \theta_{1} )S_{1} - G - \theta_{1} S_{2} + \theta_{1} w} \right] + (1 - p)\left[ {(1 - \theta_{2} )S_{1} - \theta_{2} S_{2} + \theta_{2} w} \right]$$

2 $$u_{12} = p{\mkern 1mu} \left( { - \theta_{1} S_{2} } \right) + \left( {1 - p} \right){\mkern 1mu} \left( { - \theta_{2} S_{2} {\mkern 1mu} } \right)$$

3 $$\overline{u}_{1} = qu_{11} + \left( {1 - q} \right)u_{12}$$

When the assembled steel plant chooses smart management, the expected benefit is *u*_21_, and the expected benefit of traditional management is *u*_22_, so the average benefit is *ū*_2_. The corresponding expression is as follows:

4 $$u_{21} = q\left( {R_{1} - C_{1} + G - \theta_{1} D - \theta_{1} w} \right) + \left( {1 - q} \right)\left( {R_{1} - C_{1} - \theta_{1} D} \right)$$

5 $$u_{22} = q\left( {R_{2} - C_{2} - \theta_{2} D - \theta_{2} w} \right) + \left( {1 - q} \right)\left( {R_{2} - C_{2} - \theta_{2} D} \right)$$

6 $$\overline{u}_{2} = pu_{21} + \left( {1 - p} \right)u_{22}$$

The replication dynamics equation is a continuous deterministic equation based on game dynamics, which is used to describe the evolution of population strategies over time. In the game process, the game participants will continuously adjust and optimize their strategies through learning and imitation. Based on the basic principle of replication dynamics, *q* and *p* are expressed as functions of time *t*. And based on Eqs. (1)–(3) and (4)–(6), the replication dynamics equations of government and assembled steel plants can be obtained as follows:

7 $$\begin{gathered} F\left( q \right) = \frac{dq}{{dt}} = q\left( {1 - q} \right)\left( {u_{11} - \overline{u}_{1} } \right) \\ = q{\mkern 1mu} \left( {1 - q} \right){\mkern 1mu} \left\{ {\left( {1 - {\mkern 1mu} \theta_{2} } \right)S_{1} + \theta_{2} {\mkern 1mu} w + p\left[ {\left( {S_{1} {\mkern 1mu} - w} \right)\left( {\theta_{2} - {\mkern 1mu} \theta_{1} } \right) - G} \right]} \right\} \\ \end{gathered}$$

8 $$\begin{gathered} F\left( p \right) = \frac{dp}{{dt}} = p\left( {1 - p} \right)\left( {u_{21} - \overline{u}_{2} } \right) \\ = p{\mkern 1mu} \left( {1 - p} \right)\left\{ {C_{2} - C_{1} + R_{1} - R_{2} + {\text{D}}\left( {\theta_{2} - {\mkern 1mu} \theta_{1} } \right) + {\mkern 1mu} q\left[ {G + w\left( {\theta_{2} - {\mkern 1mu} \theta_{1} } \right)} \right]} \right\} \\ \end{gathered}$$

## Model solution and analysis

### Strategic stability analysis of the government

According to the stability theorem of differential equation, the stable state of the probability that the government selects the incentive must satisfy *F*(*q*) = 0 and *F′*(*q*) < 0. The derivative of the government's replication dynamic Eq. (7) can be obtained:

9 $$F^{\prime}\left( q \right) = \left( {1 - 2q} \right){\mkern 1mu} \left\{ {\left( {1 - {\mkern 1mu} \theta_{2} } \right)S_{1} + \theta_{2} {\mkern 1mu} w + p\left[ {\left( {S_{1} {\mkern 1mu} - w} \right)\left( {\theta_{2} - {\mkern 1mu} \theta_{1} } \right) - G} \right]} \right\}$$

When $p = p^{*} = \frac{{\left( {1 - {\mkern 1mu} \theta_{2} } \right)S_{1} + \theta_{2} {\mkern 1mu} w}}{{G + \left( {S_{1} {\mkern 1mu} - w} \right)\left( {{\mkern 1mu} \theta_{1} - \theta_{2} } \right)}}$, *F*(*q*) ≡ 0 and *F’*(*q*) ≡ 0 can be obtained. At this time, any strategy formulated by the government is stable.

If $\left( {S_{1} {\mkern 1mu} - w} \right)\left( {\theta_{2} - {\mkern 1mu} \theta_{1} } \right) - G > 0$, when *p* > p^*^, $\left. {F^{\prime}\left( q \right)} \right|_{q = 0} > 0$, $\left. {F^{\prime}\left( q \right)} \right|_{q = 1} < 0$, *q* = 1 is in an evolutionary stable state, the government's strategy is incentive. When *p* < p^*^, $\left. {F^{\prime}\left( q \right)} \right|_{q = 0} < 0$, $\left. {F^{\prime}\left( q \right)} \right|_{q = 1} > 0$, *q* = 0 is in an evolutionary stable state, the government's strategy is no incentive.

If $\left( {S_{1} {\mkern 1mu} - w} \right)\left( {\theta_{2} - {\mkern 1mu} \theta_{1} } \right) - G < 0$, when *p* > p^*^, $\left. {F^{\prime}\left( q \right)} \right|_{q = 0} < 0$, $\left. {F^{\prime}\left( q \right)} \right|_{q = 1} > 0$, *q* = 0 is in an evolutionary stable state, the government's strategy is no incentive. When *p* < p^*^, $\left. {F^{\prime}\left( q \right)} \right|_{q = 0} > 0$, $\left. {F^{\prime}\left( q \right)} \right|_{q = 1} < 0$, *q* = 1 is in an evolutionary stable state, the government's strategy is incentive.

### Strategic stability analysis of assembled steel plants

The steady state for the selection of smart production in an assembled steel plant needs to satisfy *F*(*p*) = 0 and *F′*(*p*) < 0. The derivative of the replication dynamic Eq. (8) can be obtained:

10 $$F^{\prime}\left( p \right) = {\mkern 1mu} \left( {1 - 2p} \right)\left\{ {C_{2} - C_{1} + R_{1} - R_{2} + {\text{D}}\left( {\theta_{2} - {\mkern 1mu} \theta_{1} } \right) + {\mkern 1mu} q\left[ {G + w\left( {\theta_{2} - {\mkern 1mu} \theta_{1} } \right)} \right]} \right\}$$

When $q = q^{*} = \frac{{R_{2} - R_{1} + C_{1} - C_{2} + {\text{D}}\left( {{\mkern 1mu} \theta_{1} - {\mkern 1mu} \theta_{2} } \right)}}{{G + w\left( {\theta_{2} {\mkern 1mu} - \theta_{1} {\mkern 1mu} } \right)}}$, *F*(*p*) ≡ 0 and *F′*(*p*) ≡ 0 can be obtained. At this time, any strategy formulated by the government is stable.

According to the parameter relationship it is known that $G + w\left( {\theta_{2} - {\mkern 1mu} \theta_{1} } \right) > 0$, if *q* > q^*^, then $\left. {F^{\prime}\left( p \right)} \right|_{p = 0} > 0$, $\left. {F^{\prime}\left( p \right)} \right|_{p = 1} < 0$, at this time *p* = 1 is in an evolutionary stable state and the strategy of the assembled steel plant is to choose the smart plant strategy. If *q* < q^*^, then $\left. {F^{\prime}\left( p \right)} \right|_{p = 0} < 0$, $\left. {F^{\prime}\left( p \right)} \right|_{p = 1} > 0$, at this time *p* = 0 is in an evolutionary stable state and the strategy of the assembled steel plant is to choose the traditional plant strategy.

### Strategy stability analysis of two-sided gaming system

This part performs stability analysis on the two-dimensional system of the game. Combining Eqs. (7) and (8) gives the following evolutionary dynamical system for both sides of the game.

11 $$\left\{ \begin{gathered} F\left( q \right) = q{\mkern 1mu} \left( {1 - q} \right){\mkern 1mu} \left\{ {\left( {1 - {\mkern 1mu} \theta_{2} } \right)S_{1} + \theta_{2} {\mkern 1mu} w + p\left[ {\left( {S_{1} {\mkern 1mu} - w} \right)\left( {\theta_{2} - {\mkern 1mu} \theta_{1} } \right) - G} \right]} \right\} = 0 \\ F\left( p \right) = p{\mkern 1mu} \left( {1 - p} \right)\left\{ {C_{2} - C_{1} + R_{1} - R_{2} + {\text{D}}\left( {\theta_{2} - {\mkern 1mu} \theta_{1} } \right) + {\mkern 1mu} q\left[ {G + w\left( {\theta_{2} - {\mkern 1mu} \theta_{1} } \right)} \right]} \right\} = 0 \\ \end{gathered} \right.$$

Five equilibrium points can be obtained by solving equation system (11): *E*_1_ (0,0), *E*_2_ (0,1), *E*_3_ (1,0), *E*_4_ (1,1), *E*_5_ (q^*^, p^*^), where $p^{*} = \frac{{\left( {1 - {\mkern 1mu} \theta_{2} } \right)S_{1} + \theta_{2} {\mkern 1mu} w}}{{G + \left( {S_{1} {\mkern 1mu} - w} \right)\left( {{\mkern 1mu} \theta_{1} - \theta_{2} } \right)}}$, $q^{*} = \frac{{R_{2} - R_{1} + C_{1} - C_{2} + {\text{D}}\left( {{\mkern 1mu} \theta_{1} - {\mkern 1mu} \theta_{2} } \right)}}{{G + w\left( {\theta_{2} {\mkern 1mu} - \theta_{1} {\mkern 1mu} } \right)}}$. According to Friedman's study^42^, the stability of the evolutionary system can be obtained from the local stability analysis of the Jacobian matrix. The Jacobian matrix of the two-dimensional system is as follows:

12 $$J = \left[ {\begin{array}{*{20}c} {\frac{\partial F\left( q \right)}{{\partial q}}} & {\frac{\partial F\left( q \right)}{{\partial p}}} \\ {\frac{\partial F\left( p \right)}{{\partial q}}} & {\frac{\partial F\left( p \right)}{{\partial p}}} \\ \end{array} } \right] = \left[ {\begin{array}{*{20}c} {a_{11} } & {a_{12} } \\ {a_{21} } & {a_{22} } \\ \end{array} } \right]$$

Among them:

13 $$\left\{ \begin{gathered} a_{11} = \left( {1 - 2\,q} \right)\,\left\{ {\left( {1 - \,\theta_{2} } \right)S_{1} + \theta_{2} \,w - p\left[ {G + \left( {S_{1} - w} \right)\left( {\theta_{1} - \theta_{2} } \right)} \right]} \right\} \hfill \\ a_{12} = - q{\mkern 1mu} \left( {1 - q} \right){\mkern 1mu} \left[ {G + \left( {S_{1} - w} \right){\mkern 1mu} \left( {\theta_{1} - {\mkern 1mu} \theta_{2} } \right)} \right] \hfill \\ a_{21} = p{\mkern 1mu} \left( {1 - p} \right){\mkern 1mu} \left[ {G - w\left( {\theta_{1} {\mkern 1mu} - \theta_{2} {\mkern 1mu} } \right)} \right] \hfill \\ a_{22} = \left( {1 - 2\,p} \right)\,\left\{ {R_{1} - R_{2} + C_{2} - C_{1} - {\text{D}}\left( {\,\theta_{1} - \,\theta_{2} } \right) + q\left[ {G\, - w\left( {\,\theta_{1} \, - \,\theta_{2} \,} \right)} \right]} \right\} \hfill \\ \end{gathered} \right.$$

According to Lyapunov stability theory, a sufficient necessary condition for the game system to achieve ESS is that all eigenvalues of the Jacobian matrix have negative real parts. That is, the system stable state in the Jacobian matrix should satisfy the determinant $detJ = \lambda_{1} \lambda_{2} > 0$ and trace $trJ = \lambda_{1} + \lambda_{2} < 0$ (*λ*_1_ and *λ*_2_ are the two eigenvalues of the Jacobian matrix). Because of the instability of the mixed strategy point in the evolution process—the trace $trJ = 0$ of (q^*^, p^*^), only the local stability of the remaining four equilibrium points as pure strategy points in the Jacobian matrix is considered. By substituting the equilibrium point into the Jacobian matrix, the eigenvalue of each equilibrium point can be obtained and summarized in Table 3.

**Table 3:** Stability analysis of the equilibrium points.

Equilibrium point	$detJ$	$trJ$
$E_{1} \left( {0,0} \right)$	$KM$	$K + M$
$E_{2} \left( {0,1} \right)$	$- LM$	$L - M$
$E_{3} \left( {1,0} \right)$	$- KN$	$- K + N$
$E_{4} \left( {1,1} \right)$	$LN$	$- \left( {L + N} \right)$

In order to facilitate the subsequent analysis, the equation is simplified in this paper,

14 $$\left\{ \begin{gathered} K = \left( {1 - \theta_{2} } \right)S_{1} + \theta_{2} w \hfill \\ L = \left( {1 - \theta_{1} } \right)S_{1} - G + \theta_{1} w \hfill \\ M = R_{1} - R_{2} - C_{1} + C_{2} + D\left( {\theta_{2} - \theta_{1} } \right) \hfill \\ N = R_{1} - R_{2} - C_{1} + C_{2} + G + \left( {D + w} \right)\left( {\theta_{2} - \theta_{1} } \right) \hfill \\ \end{gathered} \right.$$

Since $\left( {1 - \theta_{2} } \right)S_{1} - \theta_{2} S_{2} + \theta_{2} w > - \theta_{2} S_{2}$, which is *K* > 0, it can be judged that *E*_1_ (0,0) must not be a stable point of the evolution system. Based on the four situations shown in Table 3, there are three parameter relationships satisfying $detJ > 0$ and $trJ < 0$.

Condition I: When *N* < 0, it is known that *M* < 0. Both sides of the game evolve to (1,0) under this condition, that is, the government chooses the incentive strategy and the assembled steel plant chooses the traditional plant strategy.

Condition II: When *L* < 0, *M* > 0, *N* > 0, (0,1) is the only stable point of the system. At this time, the government chooses the no incentive strategy and the assembled steel plants choose the smart plant strategy.

Condition III: When L > 0, N > 0, (1,1) is the only stable point of the system. Under this condition, the government chooses the incentive strategy, and the assembled steel plants chooses the smart plant strategy.

Specifically, the stability analysis results corresponding to the three parameter relationships are shown in Table 4.

**Table 4:** Stability analysis under different conditions.

Equilibrium point	Condition I			Condition II			Condition III		
	$detJ$	$trJ$	Stability	$detJ$	$trJ$	Stability	$detJ$	$trJ$	Stability
$E_{1} \left( {0,0} \right)$	$-$	Uncertain	Saddle point	$+$	$+$	Unstable point	$- / +$	$$\begin{gathered} {\text{Uncertain/}} \\ + \\ \end{gathered}$$	Saddle point/Unstable point
$E_{2} \left( {0,1} \right)$	$+ / -$	$$\begin{gathered} + / \\ {\text{Uncertain}} \\ \end{gathered}$$	Unstable point/Saddle point	$+$	$-$	ESS	$+ / -$	$$\begin{gathered} + / \\ {\text{Uncertain}} \\ \end{gathered}$$	Unstable point/Saddle point
$E_{3} \left( {1,0} \right)$	$+$	$-$	ESS	$-$	Uncertain	Saddle point	$-$	Uncertain	Saddle point
$E_{4} \left( {1,1} \right)$	$- / +$	$$\begin{gathered} {\text{Uncertain}}/ \\ + \\ \end{gathered}$$	Saddle point/Unstable point	$-$	Uncertain	Saddle point	$+$	$-$	ESS

Based on the above stabilization strategies and conditions, the following conclusions can be drawn:

①The government's subsidy policy is an important initiative to promote the manufacturing industry to achieve intelligent transformation and help traditional factories to move towards smart production. ②Regardless of whether or not the government sets up incentive strategies, plants will participate in smart production as long as the net benefits derived by assembled steel plants choosing smart plants are greater than those generated by the traditional model. In short, investors seek advantage and avoid disadvantage. Therefore, for the government, reasonable subsidy incentives can satisfy the revenue needs of assembled steel plants and help stimulate investors' participation. In addition, the setting of negative penalty mechanism is conducive to enhancing the safety awareness of steel plants, thus eliminating opportunistic behavior in the cooperation process.

### Numerical case study

Numerical simulation is helpful to directly reflect the results of theoretical research and can continuously and dynamically show the development rule of things. Therefore, numerical simulation is often used as a supplementary argument to game analysis. In order to test the effectiveness of the theoretical game model, this part is based on MATLAB to carry out numerical simulation analysis of the Fuzhou X Steel Structure Plant project. The first part of this section describes the case background of the Fuzhou X Steel Structure Plant project, and sets the parameters corresponding to the model. The second part obtains the dynamic evolution process of the decision-making behavior of each stakeholder in the initial state of the project through MATLAB simulation. The third part carries out numerical simulation on the sensitivity of the relevant parameter changes (including costs, subsidies, etc.) of the project participants in the initial state to provide case reference for the government to actively promote the intelligent transformation of the manufacturing industry and enhance the internal upgrading power of enterprises.

### Case background and parameter value setting

According to the policy of Fuzhou, assembled construction bidding requires one bid and one component plant. Therefore, the steel plant will seize the opportunity of the policy dividend "window". With the first-mover advantage of the steel structure production base and the "EPC + industrialization" bidding policy, Company X has vigorously expanded the steel structure assembly EPC business and realized the accelerated growth of the marketing contract amount.

The X Steel Structure Plant project is located in the suburb of Fuzhou City, Fujian Province, about 45 km away from the urban area of Fuzhou. The transportation is convenient, and the plant can be reached through the highway G70 or the national highway G316. The project covers Putian, Ningde, Nanping, Sanming, Quanzhou, Pingtan Comprehensive Experimental Zone and other central cities in Fujian Province. The project covers a land area of about 88 mu, and two steel structure production lines (heavy steel line and light steel line) are operated at the same time, with an annual capacity of 25,000 tons. The total project investment is about 49,956,700 yuan, with payback period of 7.38 years (including 1-year construction period) and internal rate of return of 11.52%. The capital contribution of Company X is in the form of fixed assets such as land after evaluation and Company Y is in cash. After the project is put into operation, both parties will share the dividends according to their respective equity ratios. In order to lead the development of the steel structure green ecological industry in Haixi region, the project is positioned as an industrial base of steel structure assembled building integrating research and development, design, production, manufacturing, sales and construction. The GS-Building and ME-House assembled steel structure building product systems with independent intellectual property rights of Y Company are introduced, forming a complete integrated solution. Through the project feasibility study report and relevant policy data, this paper makes theoretical assignments to the relevant parameters, which are detailed in Table 5.

**Table 5:** Initial parameter values of the evolutionary game model.

Parameters	Rationale	Value/million
$S_{1}$	The improvement of the government's credibility can help improve administrative efficiency and increase the government's influence and appeal^43^. To facilitate the model calculation, this paper quantifies the credibility and simplifies it to the amount of government benefits	20
$S_{2}$	As in *S*_1_, the loss of government credibility is simplified to the amount of the government's loss	20
$G$	According to the awards and subsidies mentioned in the document "Nine Measures on Advancing Industrial Digital Transformation" issued by Fujian Provincial Department of Finance, and considering other subsidy policies, the subsidy of this project is assumed to be 10 million RMB	10
$w$	According to the Regulations on Reporting, Investigation and Handling of Production Safety Accidents issued by the State Council of China, it is assumed that the penalty for safety accidents during the whole life cycle of the project is 60 million yuan	60
$R_{1}$	Based on the analysis of the financial evaluation indicators in the feasibility study report of the project, the average annual revenue of 217,930,400 yuan (in order to simplify the calculation, the average annual equipment depreciation cost is also counted in the revenue), excluding inflation, the revenue of the assembled steel smart plant project is 2179,304,000 yuan	2179.304
$R_{2}$	Assuming that the revenue generated by the traditional steel plant project is 0.7 times that of the smart plant, the revenue is 1,525,512,800 yuan	1525.5128
$C_{1}$	Based on the analysis of the financial evaluation indexes in the feasibility study report of the project, the total investment of the project is 49,956,700 yuan, and the average annual total expenditure cost during the operation period is 210,010,400 yuan, with the operation period of 10 years. Excluding inflation, the cost incurred by the assembled steel smart plant is RMB 215,096,700	2150.9607
$C_{2}$	Assuming that the cost of the traditional steel plant project is 0.7 times that of the smart factory, the cost is 1505.67249 million yuan	1505.67249
$\theta_{1}$	Smart plant production focuses on lean manufacturing, which is more prominent in safety production management. The probability of safety accidents is set at 10%	10 (%)
$\theta_{2}$	The probability of safety accidents in traditional steel plant production is set at 30%	30 (%)
$D$	The loss to be paid by the steel plant in the event of a safety accident on the project is RMB 60 million	60

### Stakeholder dynamic evolution

**Figure 3:**
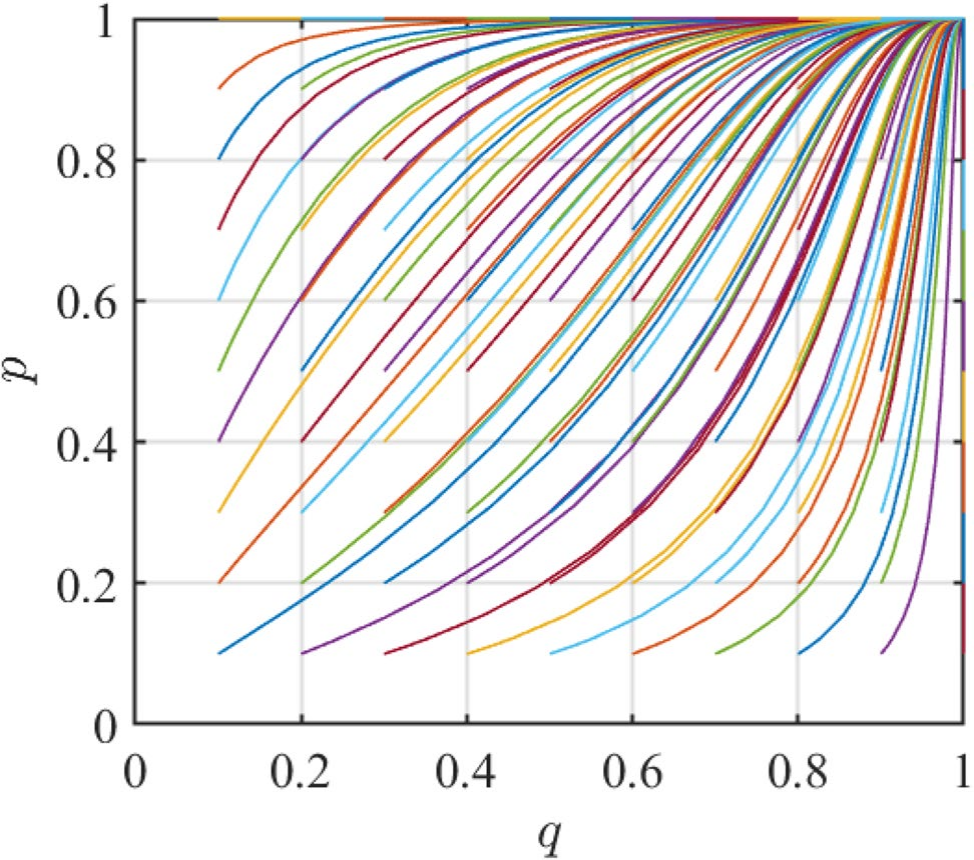
Evolutionary results of 81 times satisfying the *E*_4_ condition.

According to the initial parameter value setting of the evolutionary game model in Table 5, the parameters of the project are obtained to satisfy $\left( {1 - \theta_{1} } \right)S_{1} - G + \theta_{1} w < 0$, $R_{1} - R_{2} - C_{1} + C_{2} + G + \left( {D + w} \right)\left( {\theta_{2} - \theta_{1} } \right) < 0$. Based on MATLAB R2021a, 81 different groups of (*q*,*p*) initial strategy points can be randomly generated to test the stability of the equilibrium point *E*_4_ (1,1) in the game process. The lines with different colors in Fig. 1 represent the evolution process of 81 groups of different initial strategic points randomly produced by MATLAB in the game between the two sides. As shown in Fig. 3, as the evolution time grows, after continuous iteration, the strategies of both parties will converge to (1,1). At this time, the ESS of the government and assembled steel plants is {incentive, assembled steel smart plant}. The simulation process illustrates that the initial strategy choosing states of both sides of the game do not affect the evolutionary outcome when the constraints are satisfied. With sufficient evolutionary time, the final behavioral strategies of each stakeholder will gradually evolve into optimal solutions. Thus, the theoretical analysis in the previous section is effectively tested, while providing a basis for the initial state setting of the subsequent sensitivity analysis.

### Parameter sensitivity analysis

Sensitivity analysis, as a quantitative analysis method, aims to observe the change of the target through the adjustment of factors, so as to find out the rules. Based on the parameter setting of the case, the initial strategy of both sides of the game can be set as (*q*_0_, *p*_0_) = (0.5, 0.5). This part will simulate and analyze the relevant influencing factors in turn.

Firstly, numerical simulation is carried out for the policy subsidy limit *G*, the penalty limit *w* for safety accidents and the improvement of government credibility *S*_1_.

**Figure 4:**
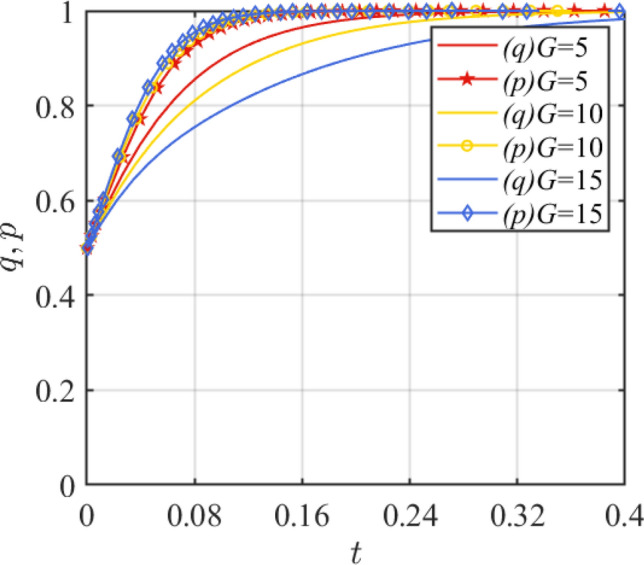
Effect of parameter *G* change.

**Figure 5:**
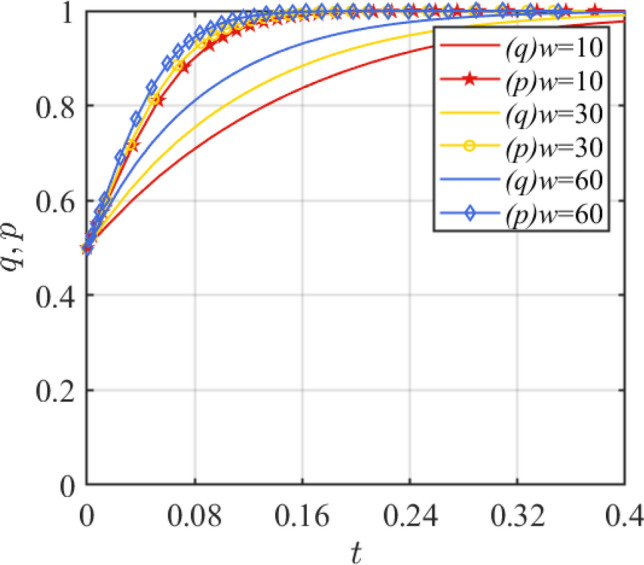
Effect of parameter *w* change.

Subsidy *G* under the government incentive strategy is set as 5, 10, and 15, respectively. Based on the two-dimensional dynamical system, the results are shown in Fig. 4. The government's punishment *w* for safety accidents is set at 10, 30, 60, and the simulation results under the two-dimensional system are shown in Fig. 5. In Fig. 4, it is shown that when *G* = 15, p evolves fastest toward 1, while q evolves slowest toward 1. This suggests that a reasonable subsidy setup can, to some extent, facilitate a win–win situation—satisfying both the revenue needs of the assembled steel plant as an investor and the government's smooth promotion of smart manufacturing initiatives. In addition, Fig. 4 also shows that excessive subsidies can cause financial burden on government departments, which leads to the tendency of disincentive. From Fig. 5, it is clear that increased government punishment for safety accidents will enhance the speed of evolution to the stability point for both sides of the game. Strict punishment mechanism is the key to promote the healthy development of smart manufacturing.

**Figure 6:**
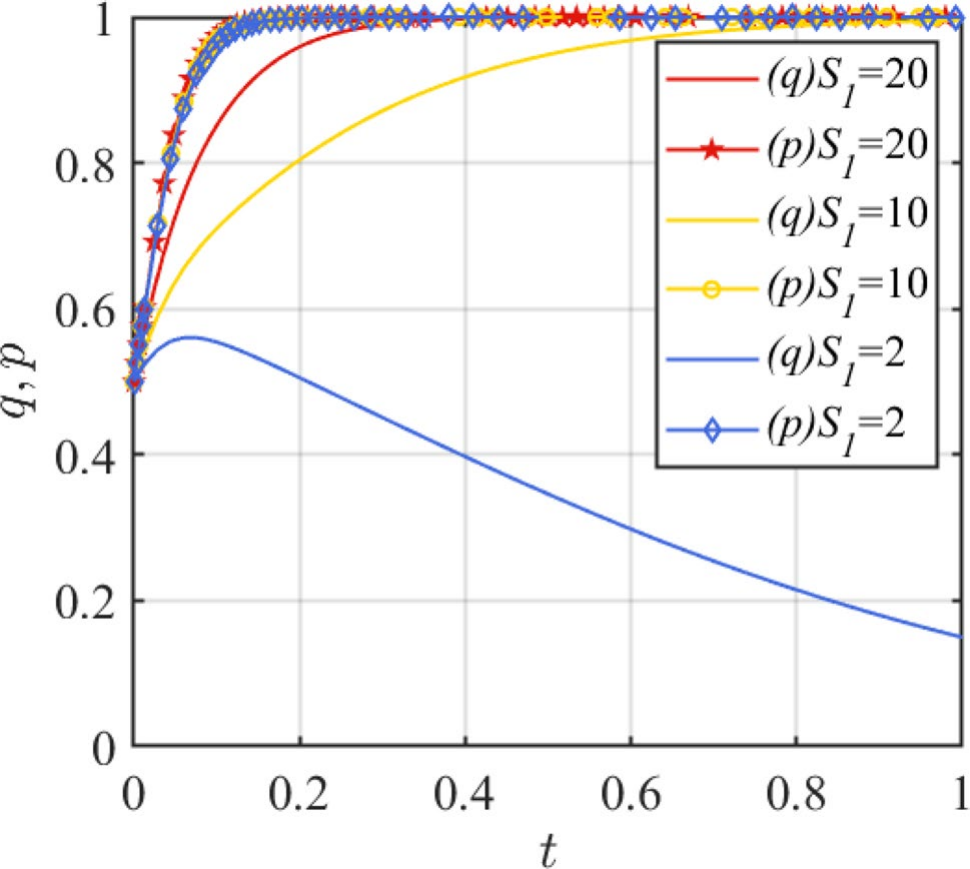
Effect of parameter *S*_1_ change.

Then, in order to analyze the impact of the improvement of government credibility on the evolution process and results, this paper will assign values of 20, 10, and 2, respectively. The simulation results of replication dynamic equations evolving over time are shown in Fig. 6. At this point, *S*_1_ in the steady state should satisfy $S_{1} > \frac{{G - \theta_{1} w}}{{1 - \theta_{1} }}$, that is, when ESS is (1,1), *S*_1_ > 4.44 (4.44 is the critical value meeting the stability conditions of (1,1), which is calculated based on the above Eq. (14) and Table 5 parameters. At this critical value, the government behaves as a horizontal line and is in a mixed strategy state. However, the change of initial value can break this state and make it evolve to pure strategy.). From Fig. 6, it can be seen that as *S*_1_ decreases, the probability of government incentives becomes lower. When *S*_1_ < 4.44, the evolutionary stable state of both sides of the game will become (0,1). As can be seen, when the project background changes, the evolutionary stability state of both sides of the game changes accordingly. Therefore, the government's incentive strategy should also be flexible to adjust with changes in project benefits, costs, safety governance capacity, and other conditions.

In order to explore the evolution of the behavioral decisions of the game subjects in the smart production model, this section conducts numerical simulations of the project's cost *C*_1_, benefit *R*_1_, the probability of a safety accident in the smart plant *θ*_1_ and the accident loss *D*.

**Figure 7:**
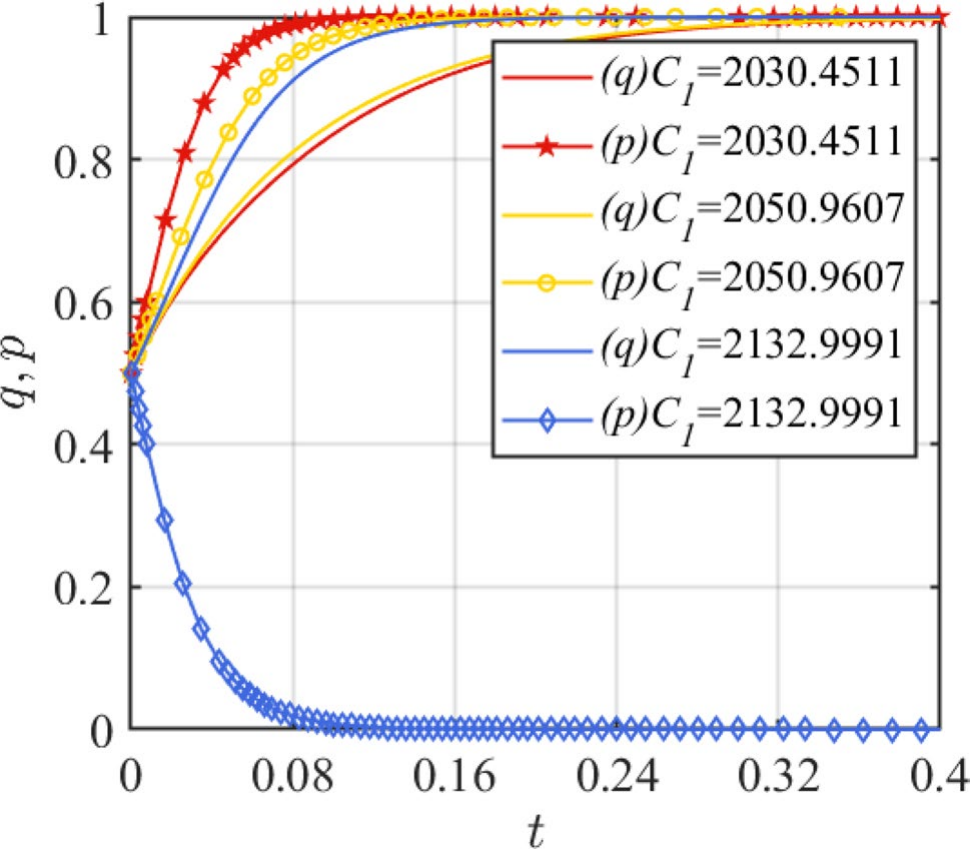
Effect of parameter *C*_1_ change.

**Figure 8:**
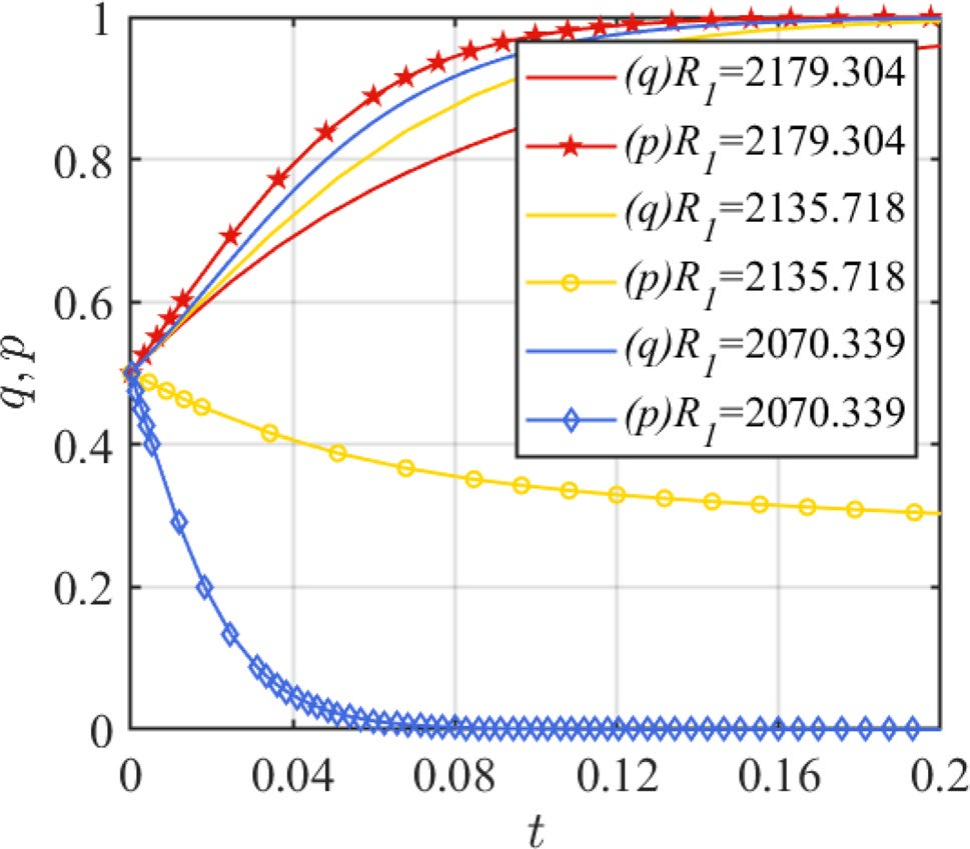
Effect of parameter *R*_1_ change.

Assign the values of *C*_1_ as *C*_1_ = 2030.4511, 2050.9607, 2132.9991, and the results are shown in Fig. 7. Assign the values of *R*_1_ as *R*_1_ = 2179.304, 2135.718, 2070.339, and the results are shown in Fig. 8. From Fig. 7, it can be seen that with the increase of the cost of assembled steel smart plant, *q* will evolve in the direction of 1, while *p* will evolve in the direction of 0. At this time, the critical value condition of *C*_1_ is $C_{1} = R_{1} - R_{2} + C_{2} + G + \left( {D + w} \right)\left( {\theta_{2} - \theta_{1} } \right)$, that is, *C*_1_ = 2193.46. This system stability state changes from (1,1) to (1,0) when the parameter change is greater than 2193.46. Figure 8 shows that the considerable benefits of the assembled steel smart plant project are the key to attracting assembled steel manufacturers to invest in it. Based on this condition, *R*_1_ satisfies $R_{1} > C_{1} + R_{2} - C_{2} - G - \left( {D + w} \right)\left( {\theta_{2} - \theta_{1} } \right)$, i.e., the critical value of *R*_1_ is 2136.8. When *R*_1_ changes less than 2136.8, the system steady state (1,1) will change to (1,0).

**Figure 9:**
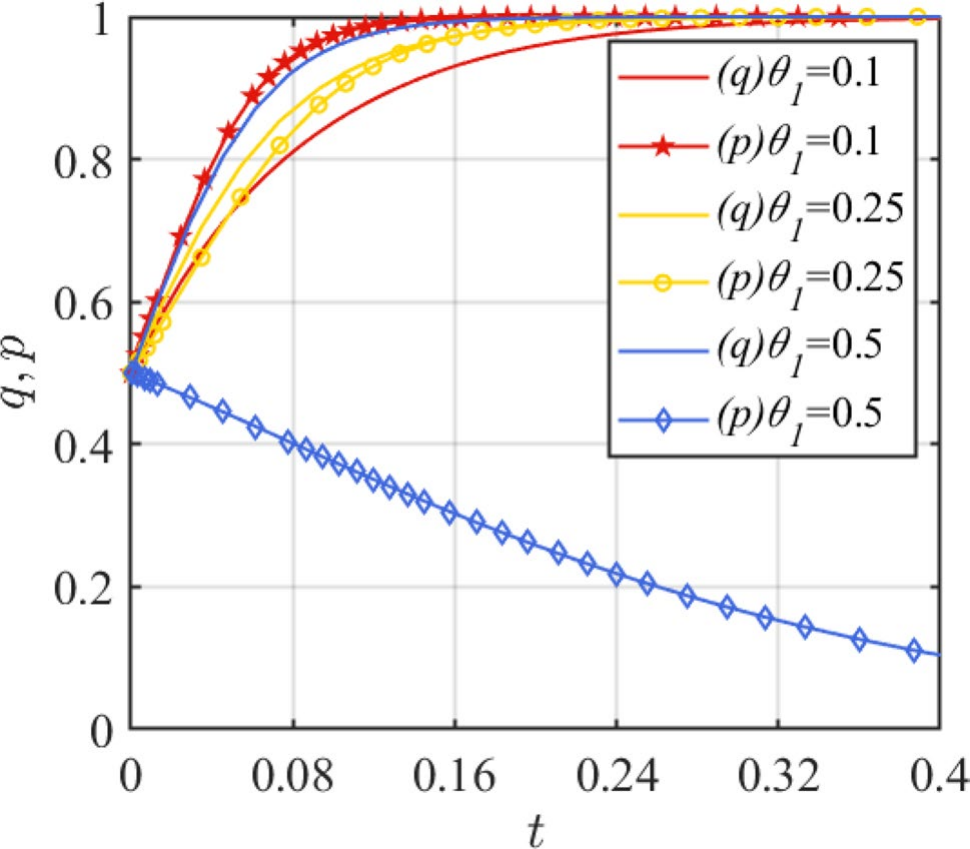
Effect of parameter *θ*_1_ change.

**Figure 10:**
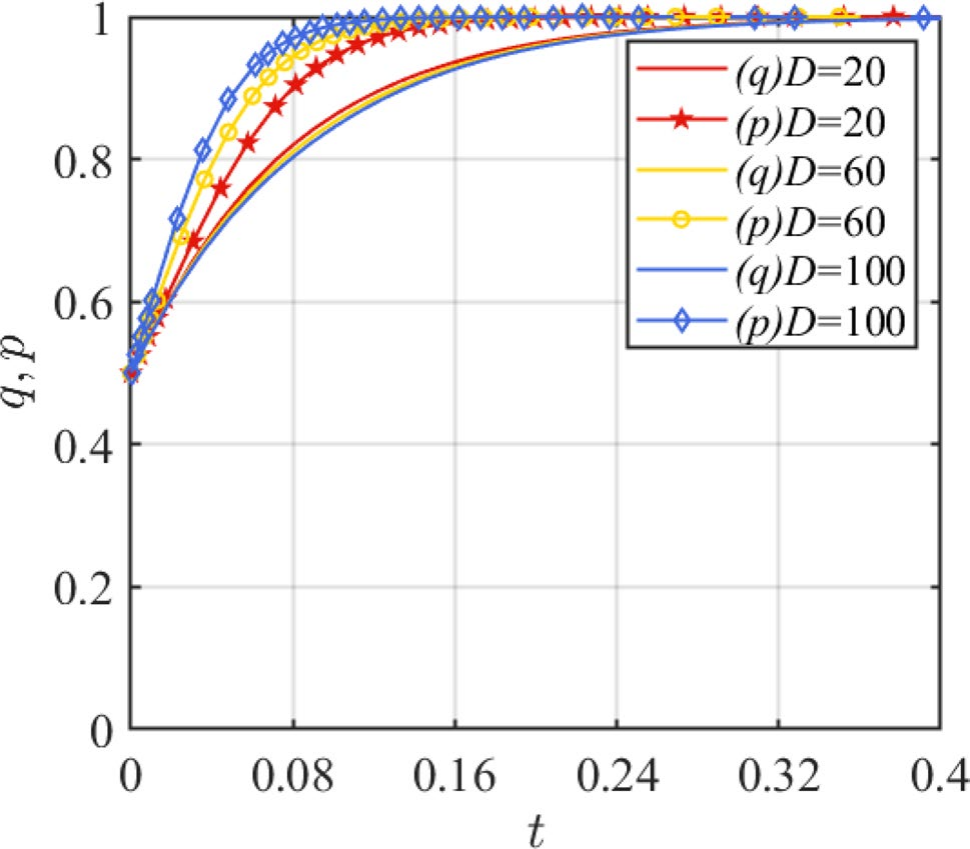
Effect of parameter *D* change.

Next, the accident probability and the amount of loss generated by the accident are analyzed. Set *θ*_1_ = 0.1, 0.25, 0.5 and the simulation results of replication dynamic equations evolution over time as shown in Fig. 9. Set *D* = 20, 60, 100 and the simulation results of replication dynamic equations evolution over time as shown in Fig. 10. Figure 9 shows that with the increase of *θ*_1_, the mood of the government to set incentives becomes stronger, and the assembled steel plant will prefer the traditional production mode. When $\theta_{1} > \frac{{R_{1} - R_{2} - C_{1} + C_{2} + G}}{D + w} + \theta_{2}$, i.e., *θ*_1_ > 0.45419, the assembled steel plant will change the original strategy. Figure 10 shows that as the loss amount *D* increases, the assembled steel plant prefers to choose the smart production option.

## Discussion

Based on the numerical simulation analysis, it can be seen that there is a stable equilibrium point (1,1) for the Fuzhou X Steel Structure Plant project. The results of sensitivity analysis show that in the process of government promoting smart manufacturing safety production, the strategy selection of game players will respond to the changes of other participants' strategies. Among them, the government incentive smart information platform to build subsidies and safety accident punishment mechanism, the assembled steel smart plant project income and the amount of safety accident losses to pay are positively correlated with the assembled steel plant to choose the smart production strategy. The cost of a smart plant project and the probability of a safety incident are negatively correlated with the probability of choosing a smart production strategy.

Based on the case numerical simulation, it can be seen that the expected cost of the project cannot be higher than 2193.46 million, while the expected revenue of the project cannot be lower than 2136.8 million. In order to obtain considerable revenues, it is necessary for the smart plant to prepare for the project site selection, project service objects and project radiation radius analysis in the pre-construction period. The policy incentive subsidy is equivalent to the net benefit of assembled steel plant, and the policy can enhance the motivation of producers to make intelligent transformation. For example, the government may set up a feasibility gap subsidy based on the project's revenue. However, in order to avoid the loss of social welfare caused by over-subsidization, replacing subsidies with rewards might be an incentive model that could be widely promoted. In addition, based on the sensitivity analysis of the accident rate and the amount of loss, when the accident rate of the smart plant in the case is high, i.e., more than 45.419%, the assembled steel plant will still choose to produce based on the traditional model. Moreover, for different smart plant projects, the stability state of game evolution will be different due to the differences in project revenue and local financial subsidies. But parameter variations can affect the entire two-dimensional dynamical system, so the evolutionary conclusions proposed in this paper are also applicable to other stability states. For the government, the incentive strategy can be adjusted appropriately according to the different evolutionary stability states, so as to promote the positive development of the government-enterprise cooperation system.

## Conclusion

Compared with the traditional steel production mode, the introduction of the smart plant has brought about a change in the safety production management mode. The main breakthrough points are reflected in the following three aspects: (1) safety production intelligence. In the assembled steel smart plant, except for a small number of supervisors, the production chain is mechanized and automated. Smart factories use a lot of machinery for production, which improves the potential benefits of enterprises while freeing up labor. (2) Safety system integration. Based on IoT technology, basic data connectivity can be formed within the plant to achieve timely data utilization. At the same time, the establishment of the security database is an important guarantee of the core secrets of the plant. The process of linking data in the smart factory is supplemented by a security system for supervision and real-name system of data use is implemented, which can effectively guarantee the security of factory data. (3) Clustering of teamwork. The enhancement of the safety system does reduce the number of personnel, but it does not completely eliminate the need for people; On the contrary, people are still the core of the new safety management system of the assembled steel smart plant. The assembled steel smart plant enhances the intelligence of the plant by stimulating and releasing human creativity with the help of mechanisms such as learning, feedback and intergenerational exchange.

Based on the perspective of safety production control, this paper discusses the management status of assembled steel production industry, and establishes an evolutionary game model between government and assembled steel plants. Through game model solution and case numerical simulation analysis, the following conclusions can be drawn: compared with traditional models, intelligent management has a positive impact on the improvement of enterprises' long-term benefits, and with the help of intelligent management tools such as big data, the probability of safety accidents can be effectively reduced. The production management strategy selection of assembled steel plants is influenced by the government's incentive subsidy mechanism, punishment mechanism, income and cost generated by traditional/intelligent management, the probability of safety accidents and accident losses and other factors. Inevitably, the introduction of advanced technology and equipment will lead to an increase in costs. Therefore, the strategic evolution of manufacturers is closely related to the government's emphasis on production safety and incentive measures. Positive incentives (i.e., subsidy mechanism) and negative incentives (i.e., punishment means) of policies should complement each other: on the one hand, the subsidy mechanism can alleviate the financial pressure of the assembled steel structure production industry to enter the smart plant field and reduce the entry threshold. On the other hand, the safety accident punishment mechanism helps to strictly control the safety production management of the industry, promote the transformation of the industry to smart production management mode, and reduce the probability of safety accidents.

In this paper, the technical cost and safety efficiency improvement at each stage of smart factory operation are not divided in detail. In the future research, the classified production cost can be further refined, and the probability of safety accidents can be predicted based on the simulation method, so as to provide more specific suggestions for the selection of smart production mode and the design of policy incentive mechanism in the assembled steel structure industry.

## Data Availability

All data generated or analyzed during this study are included in this published article.
